# Linkage analysis of the simulated data – evaluations and comparisons of methods

**DOI:** 10.1186/1471-2156-4-S1-S70

**Published:** 2003-12-31

**Authors:** Swati Biswas, Charalampos Papachristou, Mark E Irwin, Shili Lin

**Affiliations:** 1 Department of Statistics, The Ohio State University, 1958 Neil Avenue, Columbus, Ohio, USA

## Abstract

The goal of this study is to evaluate, compare, and contrast several standard and new linkage analysis methods. First, we compare a recently proposed confidence set approach with MAPMAKER/SIBS. Then, we evaluate a new Bayesian approach that accounts for heterogeneity. Finally, the newly developed software SIMPLE is compared with GENEHUNTER. We apply these methods to several replicates of the Genetic Analysis Workshop 13 simulated data to assess their ability to detect the high blood pressure genes on chromosome 21, whose positions were known to us prior to the analyses. In contrast to the standard methods, most of the new approaches are able to identify at least one of the disease genes in all the replicates considered.

## Background

We consider various standard as well as new linkage analysis tools and methods. The Genetic Analysis Workshop 13 simulated complete data set gives an excellent opportunity to evaluate and compare these methods. First, we compare the standard nonparametric approach for sib-pair analysis as implemented in MAPMAKER/SIBS [1] with an extension of a recent confidence set approach [2]. We expect the latter to perform better because it does not require multiplicity adjustment for the number of tests executed. Next, we assess the performance of a new Bayesian parametric approach for detecting linkage that takes into account possible locus heterogeneity. Another issue of interest is how much linkage information can be gained if the large pedigrees can be analyzed as a whole compared with analyzing reduced ones, a practice adopted by GENEHUNTER [3]. To this end, we compare the GENEHUNTER results with a new software package, SIMPLE [4], that can handle both large pedigrees and a moderate number of loci.

## Methods

### Phenotype definition, choice of genomic regions, and selection of replicates

We focus on a qualitative trait, high blood pressure. In addition to making use of the binary variable in the data, we also take into consideration several other factors. First, there is a related variable, hypertensive treatment, which is confounded with the presence or absence of high blood pressure. So, we combine observations on these two variables. Second, we have observations on each person over a range of time; this longitudinal feature of the data needs to be considered. Further, we note that by a certain (old) age, most people develop high blood pressure. This is most likely not attributable to a genetic effect. Also, a person may have one or more *isolated *observation(s) of high blood pressure or hypertensive treatment just by chance, e.g., if it happens to be a very stressful period for that person. That person should not be considered as (genetically) affected. Taking into account of all these factors, we label the following people as affected: a Cohort 1 person if he/she has three consecutive events, or a Cohort 2 person if he/she has two consecutive events, where event is defined as "high blood pressure or hypertensive treatment at or before the age of 55". Note that the difference in criteria for the two cohorts is due to the fact that the period between three consecutive events in Cohort 1 is roughly the same as two consecutive events in Cohort 2.

Instead of performing a whole-genome scan, we focus on chromosome 21, which has three genes, b37, s10, and s12, that directly affect high blood pressure. We note that the two high blood pressure genes, b37, and s12, are very close to each other. So we treat them as one gene and refer to it as s12. We compare and contrast methods in terms of their power and precision for finding these genes. We also study chromosome 20 to assess false-positive rates because it does not have any disease genes.

For the confidence set approach, we randomly select five replicates from the first batch of 25 replicates of the simulated data, which yield estimated risk characteristics compatible with a single-locus model. For the Bayesian approach we analyze four randomly chosen replicates, one from each of the four batches provided. Because SIMPLE is computationally intensive, we analyze only one replicate for the comparison between GENEHUNTER and SIMPLE. To further save time, this replicate is randomly chosen from the four replicates used in the Bayesian approach because this method utilizes GENEHUNTER to compute LOD scores. We do not intend to make comparisons among the three new methods, as they are based on different types of data, and/or different model assumptions. Thus, we do not necessarily analyze the same replicates for all new methods.

### MAPMAKER/SIBS [1] and GENEHUNTER [3]

We use these popular standard packages for exact LOD score and IBD calculations. Both are based on the hidden Markov model approach.

### Confidence set approach [2]

This approach gives a confidence set of markers (leading to confidence intervals) for the locations of disease genes. We consider this method here in a nonparametric setting. The essence of this method lies in a new (non-traditional) formulation of linkage hypotheses. For each marker *m*, the hypotheses are *H*_*om*_: θ_*m *_≤ θ_*o *_versus *H*_*am*_: θ_*m *_> θ_*o*_, where θ_*m *_is the recombination fraction between the disease and marker loci. We set θ_*o *_to be the recombination fraction corresponding to a genetic distance of 10 cM, which is roughly half of the largest distance between any two adjacent markers in the simulated data. Note that this is a two-point approach. But the above formulation of hypotheses renders multiplicity adjustment unnecessary, no matter how many tests are performed. This approach needs population risk characteristics (population prevalence, risks of various types of relatives), which we estimate from the sample itself. We apply this approach to affected sib-pair (ASP) data, both with and without parental data. These results are compared with those from MAPMAKER/SIBS.

### Bayesian approach accounting for heterogeneity

We describe this approach for the situation in which we assume there is only one disease gene on the chromosome of interest. Suppose there are *k *families in the sample. Let α_*i *_= P(disease causing gene of the *i*^th ^family is linked to the marker map), *i *=1,..., *k*, and let *d *be the position of disease gene on the chromosome. We formulate the mixture likelihood of the sample following Ott [5], but with each family having its own heterogeneity parameter, α_*i*_. We assume that the prior distributions of α_*i *_values are independent *U*(0,1). GENEHUNTER is used for the calculation of usual LOD scores at several distances, labelled 1 through *N*, on the chromosome. The prior distribution of *d *is defined over {1,..., *N*, ∞} with probabilities 1/(22*N*) for the first *N *points and 21/22 for ∞. This approach accounts for heterogeneity (if any) but is applicable to homogeneous samples as well.

We use Markov chain Monte Carlo (MCMC) methods to sample from the posterior distributions of α_*i *_values and *d*. The chain is run for a burn-in period of 10,000 iterations, followed by an additional 90,000 iterations. The posterior distributions are estimated based on these 90,000 iterations. The proportion of the non-infinity distances, , can be interpreted as an estimate of the posterior probability for linkage, *p*. Hence, a value of  greater than 0.5 can be taken as an initial signal of linkage at that chromosome. In that case, the mean of the non-infinity distances, , gives an estimate of the position of disease gene. This approach has also been extended to the situation of two disease genes on a chromosome. With this extension, we want to see if we could detect the two genes on chromosome 21 simultaneously. We also evaluate our approach under various models to examine the effect of model specification.

### SIMPLE [4]

SIMPLE is a Monte Carlo method based on sequential imputation and makes use of *all *available information on *all *pedigree members. We compare the S_pairs _scores from SIMPLE with those from GENEHUNTER.

## Results

Before presenting the results, we note the following for ease in making comparisons. On chromosome 21, there are six markers. We use the sex-averaged distances in our analysis. The disease gene s12 lies between markers 3 (26.56 cM) and 4 (40.02 cM) at position 29.46 cM. The other gene, s10, is located at 53.59 cM between markers 5 (43.89 cM) and 6 (63.35 cM).

### MAPMAKER/SIBS versus confidence set approach

This analysis is performed on five replicates. Table 1 summarizes the results for chromosome 21 when no parental information is used. We report 95% confidence intervals for the locations of disease genes. For MAPMAKER/SIBS, we present the positions of the modes of the curve (if any) that exceed at least one of the two cutoffs, 2.33 and 3.09, corresponding to the nominal levels of 0.01 (suggestive evidence) and 0.001 (significant evidence), respectively [1].

From Table 1, we see that for the confidence set method, the gene s12 is included in the 95% confidence intervals for three replicates while the gene s10 is included for two replicates. The confidence intervals for Replicate 6 include both s10 and s12. Also, Replicates 9, 13, and 18 miss either one or two disease genes by at most a few centimorgans. In contrast, MAPMAKER/SIBS does not detect any disease genes in two replicates with the cut-off 2.33. With the more stringent criterion (cut-off 3.09), only one out of the five replicates detects a signal for linkage.

For chromosome 20, the confidence set approach gives one false positive for Replicate 9. There are no other false positives by either method.

### Bayesian approach accounting for heterogeneity

This method is applied to Replicates 1, 32, 54, and 85. The chromosome 21 results for Replicate 54 are shown in Table 2. The table shows the marginal analysis (single disease gene on a chromosome) as well as the joint analysis (two disease genes on a chromosome) for eight models. The first two models are the kind of incomplete penetrance models one might use as an approximation to the true but unknown complex model. The other six models correspond to the models of s12 and s10 genes. For each of these two genes we use the three models corresponding to the systolic blood pressure (SBP), diastolic blood pressure (DBP), and their average.

Table 2 shows that there is a strong signal of linkage for all models except for the joint analysis under Model 6, which gives a weak signal at the first location. All the means from marginal analysis place the single disease gene between loci 4 and 5. The two means in the joint analysis place the two disease genes between loci 3 and 4 and loci 5 and 6, the actual locations of s12 and s10, respectively. It seems that in the marginal analysis, because of the combined effect of two genes, the single gene location is indicated at a position that is in between the two actual positions. In the other three replicates, there is a strong signal of linkage between loci 5 and 6, i.e., for gene s10, but none of them showed signal for s12. This is seen for both the marginal and the joint analysis.

Marginal analysis on chromosome 20 shows only one false positive (out of a total of 32 analyses) for the second model in Replicate 1. Since overall there is no signal of linkage found in the marginal analysis, it is doubtful that any joint analysis would yield positive result and hence no further analysis is carried out.

### GENEHUNTER versus SIMPLE

**Figure 1:**
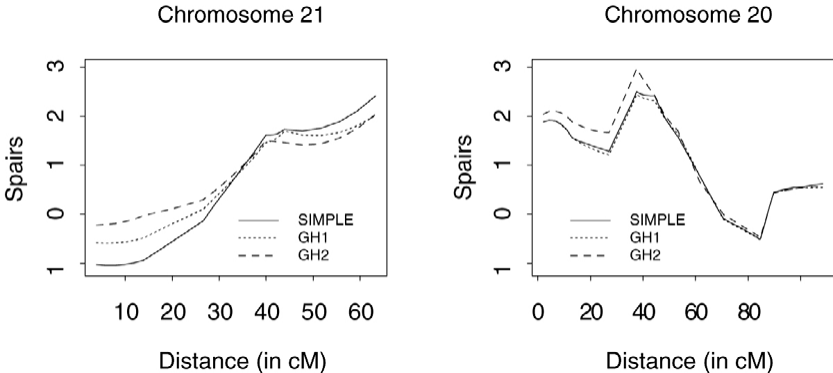
**S_pairs _scores using GENEHUNTER (GH) and SIMPLE for chromosome 21 and chromosome 20 **GH1: GH scores with skipped pedigrees; GH2: GH scores without skipped pedigrees.

We consider Replicate 85 for this comparison. For this replicate, GENEHUNTER drops individuals in 23 pedigrees. It also skips 27 pedigrees (15 of these pedigrees are informative for linkage) because these pedigrees become disconnected after GENEHUNTER drops individuals. In contrast, SIMPLE can analyze all pedigrees in their entirety. We use SIMPLE to calculate the S_pairs _statistics of the pedigrees that GENEHUNTER skips or reduces, and add them to the S_pairs _scores of the pedigrees that GENEHUNTER analyzes without reduction. These are the total scores that SIMPLE would provide estimates for, if we analyze all pedigrees using it. We plot these scores in Figure 1 (solid curve). In the same figure, the dash curve corresponds to the scores given by GENEHUNTER automatically, i.e., they do not include the scores of the pedigrees that GENEHUNTER skips. Further, we manually drop individuals in the 15 informative pedigrees skipped by GENEHUNTER and recalculate the scores given by GENEHUNTER after including them. The dotted curve in the figure is of those scores. Although the process of manually dropping individuals from each pedigree such that it remains connected is extremely labor intensive, we did it to make the comparison between GENEHUNTER and SIMPLE more fair. We see that, for chromosome 21, SIMPLE gives an S_pairs _score of 2.42, which exceeds the customary threshold of 2.33 for suggestive linkage [1]. On the other hand, S_pairs _scores for GENEHUNTER, even after including the skipped pedigrees, do not exceed the same threshold. For chromosome 20, all the three curves indicate a false positive.

## Discussion

The confidence set approach based on ASP data is more successful in identifying the disease genes in the replicates considered compared with the standard method. An attractive feature of this approach is that no multiplicity adjustment is needed. Also, unlike the traditional method, a confidence interval with known properties can be deduced. The reported confidence intervals are wide, though, due to the nature of single marker analyses. The results obtained by including parental information are similar.

The Bayesian approach incorporating heterogeneity is able to identify s10 in all replicates and s12 in one out of four replicates. We note that we could have gained more linkage information by using SIMPLE to calculate LOD scores without dropping individuals. If the marginal analysis shows a signal for linkage, it seems worthwhile to explore the joint analysis. We note that the method used to simulate the data does not lead to a genetically heterogeneous sample. This is reflected in the α_*i *_values that are all close to 0.5 in the marginal analysis. It shows that none of the families are *clearly *linked or unlinked. It is encouraging to see that our approach yields positive results in this situation. Further we see that this method is reasonably robust to model specification as all the models we considered give similar results. Nevertheless, this procedure needs further evaluation and refinement.

Comparison of SIMPLE with GENEHUNTER shows that by being able to handle large pedigrees as a whole, SIMPLE gives higher scores at positions in the vicinity of the disease locus. Since these results are based on a single replicate only that does not warrant general conclusions, we refer interested readers to the extensive simulation study that shows considerable power gains by using SIMPLE [4].

In our search for disease genes, we identify s10 in a greater number of replicates than s12. This indicates that the effect of s10 is much greater than s12, consistent with the simulation model. We note that only the new methods are able to identify the gene s12. So, overall, our methods seem to be promising in the sense that there is an evidence of power gain without increasing false-positive rates considerably. However, we caution any generalization of these results, as the number of replicates studied is relatively small.

## Figures and Tables

**Table 1:** Results using confidence set approach (CS) and MAPMAKER/SIBS (MM).

	**Replicate**				
	
**Method**	**6**	**9**	**13**	**18**	**25**
**CS**	(16.6,36.6) ∪ (53.4,73.4)	(16.6,50)	(30,50.2)	(30,73.4)	(0,36.5)
**MM1^a^**	63.4	None	None	43.9 & 63.4	63.4
**MM2^b^**	None	None	None	None	13.8

^a^MM1: Positions with scores ≥ 2.33 but ≤ 3.09. ^b^MM2: Positions with score ≥ 3.09.

**Table 2:** Bayesian approach result for Replicate 54

	**Model**				**Marginal^c^**		**Joint**			
			
No.	Freq.^a^	Penetrances^b^					_1_	_1_	_2_	_2_
1	0.2	0.05	0.50	0.70	1.00	42.2	0.92	38.5	0.92	46.3
2	0.1	0.10	0.50	0.70	1.00	42.3	0.85	37.8	0.90	46.4
3	0.2	0.00	0.20	0.50	1.00	42.4	0.89	39.2	0.92	48.3
4	0.2	0.00	0.50	0.60	1.00	41.7	0.85	36.9	0.92	45.4
5	0.2	0.00	0.35	0.55	1.00	41.8	0.81	38.2	0.92	46.3
6	0.3	0.00	0.60	1.00	1.00	42.9	0.51	30.7	0.98	45.6
7	0.3	0.00	0.20	0.60	1.00	42.6	0.96	39.7	0.93	48.6
8	0.3	0.00	0.40	0.80	1.00	42.4	0.95	39.2	0.95	48.2

^a^Freq., P(D), where D is disease allele. ^b^Penetrances are for genotypes dd, Dd, DD, in that order. ^c ^ and  represent the estimated posterior probability of linkage and the corresponding position of the disease gene for the marginal analysis. Similar notations for joint analysis are used.
